# Monitoring lung and cerebral oxygenation using near-infrared spectroscopy in preterm infants during kangaroo mother care

**DOI:** 10.1007/s00431-024-05674-5

**Published:** 2024-08-09

**Authors:** Carlo Dani, Silvia Perugi, Camilla Pizzetti, Chiara Poggi, Iuri Corsini, Simone Pratesi

**Affiliations:** 1 Division of Neonatology, Careggi University Hospital of Florence, Largo Brambilla, 3, Florence, 50141 Italy; 2 Department of Neurosciences, Psychology, Drug Research and Child Health, University of Florence, Florence, Italy

**Keywords:** Lung oxygenation, Kangaroo mother care, Near-infrared spectroscopy, Preterm infants

## Abstract

Lung function has never been assessed during kangaroo mother care (KMC) in preterm infants. We measured lung (rSO_2_L) and cerebral (rSO_2_C) oxygenation by near-infrared spectroscopy (NIRS) in infants born at less than 32 weeks of gestation or weighing ≤ 1500 g during KMC. rSO_2_L, rSO_2_C, and pulmonary (FOEL) and cerebral (FOEC) tissue oxygen extraction fraction were measured in 20 preterm infants before, during, and after a 2-h period of KMC at a mean postnatal age of 36 ± 21 days of life. We found that rSO_2_L, rSO_2_C, FOEL, and FOEC did not change in our patients. After 120 min of KMC, rSO_2_L was lower (71.3 ± 1.4 vs. 76.7 ± 4.6%; *P* = 0.012) in infants with BPD (*n* = 6; 30%) than in infants without BPD (*n* = 14 = 60%), while FOEL was higher (0.26 ± 0.02 vs. 0.20 ± 0.05; *P* = 0.012).

*Conclusion*: Cerebral and lung oxygenation did not change in preterm infants during KMC. A transient decrease in lung oxygenation was offset by the increase in oxygen extraction, but these changes were clinically insignificant. These results confirm the safety of KMC in preterm infants who are in stable clinical conditions.

**Table:** 

What is Known
*• Kangaroo mother care (KMC) is widely used to improve the care of preterm newborns since it improves their outcome.*
*• KMC is safe as patients’ vital parameters, are not negatively affected, but lung function has never been directly assessed.*

What is New
*• Cerebral and lung oxygenation measured by near-infrared spectroscopy did not change during KMC.*
*• A transient decrease in lung oxygenation compensated for by the increase in oxygen extraction occurred only in infants with BPD, but these changes were clinically insignificant.*

**Supplementary Information:**

The online version contains supplementary material available at 10.1007/s00431-024-05674-5.

## Introduction

Kangaroo mother care (KMC) has been widely used to improve the care of preterm newborns [1–4]. Although KMC has not been associated with a decrease in mortality in low birth weight infants, it has proved to be effective in reducing the risk of late-onset sepsis and the length of hospital stay, increasing the frequency of breastfeeding at discharge, and improving growth in weight, length, and head circumference [5]. KMC has also been reported to improve neurophysiological development and brain volume growth in very preterm infants [6, 7]. Other important well-recognized advantages of KMC are the positive psychological effects on parents, mother/father-child bond [8], and parental self-esteem [4].

Several studies have investigated the physiological stability of preterm newborns during KMC and it has been demonstrated that it is safe as most important parameters—heart and respiratory rate, body temperature, fraction of inspired oxygen (FiO_2_), pulse oximetry (SpO_2_), and regional cerebral oxygen saturation (rSO_2_C)—are not negatively affected [1]. In fact, clinically stable very low birth weight infants are able to maintain stable cerebral oxygenation in different positions [9], including the head-up position relative to the rest of the body tilted assumed by newborns during KMC [10]. Lorenz et al. have found that KMC does not influence cerebral oxygenation and other physiological parameters in preterm infants requiring invasive and non-invasive respiratory supports [11]. However, some concerns have been raised regarding the respiratory activity of these patients during KMC, as their lung function has never been directly assessed [12, 13].

We have recently demonstrated that near-infrared spectroscopy (NIRS) allows continuous non-invasive monitoring of regional lung oxygenation (rSO_2_L) in premature infants providing a useful point-of-care tool for assessing lung function [14].

Based on these considerations, we hypothesized that rSO_2_L does not worsen during KMC and, to assess this hypothesis, we planned this prospective observational study in a cohort of preterm infants in stable clinical conditions in whom lung oxygenation was measured by NIRS.

## Methods

### Patients

This prospective observational study was carried out at the third level Neonatal Intensive Care Unit (NICU) of the Careggi University Hospital of Florence after approval by the Pediatric Ethics Committee of Tuscany. Infants with gestational age < 32 weeks or birth weight < 1500 g and with a postnatal age > 7 days of life were enrolled in the study, after informed parental consent, if deemed suitable for KMC. Suitability for KMC was decided on the basis of good clinical condition and stability of vital parameters (body temperature, heart and respiratory rate, systemic blood pressure, SpO_2_ > 90% with FiO_2_ < 40%, absence of episodes of apnea in the previous 6 h). Exclusion criteria were major congenital malformations, chromosomal anomalies, intraventricular hemorrhage > 1^st^ degree, and sepsis.

### Study design

Enrolled patients were studied with NIRS (Root®Masimo Corporation, Irvine, CA, USA) for the measurement of rSO_2_L and rSO_2_C starting 30 min before the start of KMC and ending 60 min after its interruption, with a sampling interval of 6 s. rSO_2_ measurements obtained using NIRS technique reflect a combination of venous, arterial, and capillary oxygenated/deoxygenated intravascular hemoglobin in a ratio of approximately 75:20:5 [15].

### Kangaroo mother care

For the purpose of this study, the duration of KMC was 2 hours (± 20 min). We chose to standardize the duration of KMC to limit possible biases due to shortening or prolongation. Mothers were seated in a reclining chair at a 60° angle wearing an open front blouse. Infants were placed naked, except for a diaper and hat, directly on the skin between the breasts and covered with a light blanket. Infants were fed 1 h before KMC. All infants were continuously monitored by electrocardiogram and their heart rate, systemic blood pressure, SpO_2_, and body temperature were measured hourly. KMC was discontinued in the event of thermal instability, food intolerance (i.e., regurgitation/vomiting), onset of apnea/tachypnea/dyspnea/bradycardia, or increase in FiO_2_ > 10% for > 10 min to maintain an SpO_2_ > 90%.

### NIRS measurements

Near-infrared spectroscopy (NIRS) is a non-invasive tool allowing the measurement of regional tissue oxygen saturation (rSO_2_) which is the ratio between oxygenated hemoglobin and total hemoglobin. NIRS has been used in several studies to evaluate cerebral oxygenation and, to less extent, renal, hepatic, and splanchnic oxygenation [15–19]. However, although the penetration depth of 1.0–1.5 cm of the NIRS light [20] is appropriate for the study of the oxygenation of the lung parenchyma in preterm infants, this possible use has been poorly investigated [21].

Two self-adhesive optodes containing a light-emitting diode and two receiving sensors adequately spaced were applied to each patient. One will be positioned along the right mid-axillary line in correspondence with the 4^th^–6^th^ intercostal space for the measurement of rSO_2_L [14], and the other on the forehead for the measurement of rSO_2_C [22]. All measurements were taken during calm phases or during newborn sleep to reduce NIRS artifacts.

Based on the rSO_2_S, rSO_2_C, and SpO_2_ measurements, we calculated the pulmonary (FOEL) and cerebral (FOEC) fractional oxygen extraction ratio, using the formula FOE = [(SpO_2_-rSO_2_)/SpO2]. This parameter reflects the balance between oxygen supply and consumption. Therefore, an increase in FOE suggests an increase in oxygen extraction by the tissues, due to the greater consumption of oxygen in relation to its supply, while its decrease suggests a lower use of oxygen compared to the supply [23, 24].

We then calculated the cerebro-pulmonary oxygenation ratio (CPOR: rSO_2_L/rSO_2_C), the ratio between the oxygen saturation of lung tissue compared to the brain tissue. As cerebral perfusion is self-regulating while lung perfusion is not, CPOR is reduced when there is a decrease in pulmonary blood flow, whereas it remains unchanged under normal conditions.

All NIRS data were recorded 30 ± 10 (T_before_) min before KMC, 30 ± 10 (T_30min_), 60 ± 20 (T_60min_), 120 ± 20 (T_120min_) after it began, and 30 ± 10 (T_after30min_), 60 ± 20 (T_after60min_) min after its interruption together with SpO_2_. All patients were studied once only.

### Data collection

For each patient studied, we reported gestational age, birth weight, sex, type of delivery, Apgar score at 5 min, antenatal steroids, age at the start of NIRS measurements, need for non-invasive and invasive respiratory support (mechanical ventilation ), early discontinuation of KMC and reasons for discontinuation, postnatal steroids, patent ductus arteriosus requiring treatment [25], bronchopulmonary dysplasia (BPD), necrotizing enterocolitis (NEC) requiring surgical treatment, sepsis, 1^st^ degree intraventricular hemorrhage (IVH), ≥ 3^rd^ degree retinopathy of prematurity (ROP), and mortality or length of hospital stay. BPD was diagnosed and defined as mild, moderate, or severe in agreement with Jobe and Bancalari [26]. Intraventricular hemorrhage and NEC were diagnosed according to Papile [27] and Bell [28] criteria, respectively. ROP was graded according to the international classification of retinopathy of prematurity [29].

### Statistical analysis

The primary objective of the study was the measurement of changes in rSO_2_L during KMC in a cohort of preterm newborns using NIRS. Secondary objectives of the study were the measurement of changes in rSO_2_C, the calculation of pulmonary (FOEL) and cerebral (FOEC) tissue oxygen extraction fraction, and the calculation of the cerebro-pulmonary oxygenation ratio (CPOR) during KMC. Moreover, we compared NIRS variables in the subgroups of infants with or without BPD [26].

A sample size of at least 16 infants was calculated to detect a statistically significant 10% change in rSO_2_L (from 70 ± 10 to 60 ± 10%) measured before and after starting of KMC with 80% power at 0.05 level. Considering possible data loss, we planned to increase the sample size by 25% to 20 patients.

Clinical characteristics of patients will be described as mean ± SD, rate and percentage, or median and range. For each NIRS variable (rSO_2_L, FOEL, rSO_2_C, FOEC, CPOR), we calculated the mean (± SD) of selected 5-min periods which was chosen at the end of T_before_, T_30min_, T_60min_, T_120min_, T_after30min_, and T_after60min_. We made this choice to obtain maximum stability of the NIRS signal. However, sometimes this was not possible due to the occurrence of unwanted artifacts (generally caused by patient movements): in this case, the 5-min artifact-free period closest to the end of the study period was selected.

Serial measurements of studied variables were compared with repeated-measures analysis of variance (ANOVA). A *P* < 0.05 will be considered statistically significant.

## Results

Twenty infants were enrolled in the study at the mean postnatal age of 36 ± 21 days of life and rSO_2_L was measured in all of them during KMC without discontinuations and interferences with assistance. Their clinical characteristics are detailed in Tables 1 and S1.

**Table 1:** Demographic and respiratory supports at NIRS recording in preterm infants studied during kangaroo mother care (KMC). Mean (± SD) or median (range)

	Infants studied during KMC (*n* = 20)
Gestational age (wks)	28.3 ± 2.0
Birth weight (g)	1046 ± 270
Female	9
Vaginal delivery	2
Twin	4
Antenatal steroids	19
Apgar Score at 5 min	8 (7–9)
Age at NIRS recording (d)	36 ± 21
Respiratory support at NIRS recording:	
O_2_-therapy	3
HFNC	1
FiO_2_ at the beginning of the recording	0.22 ± 0.82
SpO_2_ at the beginning of the recording (%)	94.8 ± 3.3

*HFNC*, high-flow nasal cannula; *NIRS*, near-infrared spectroscopy

**Fig. 1:**
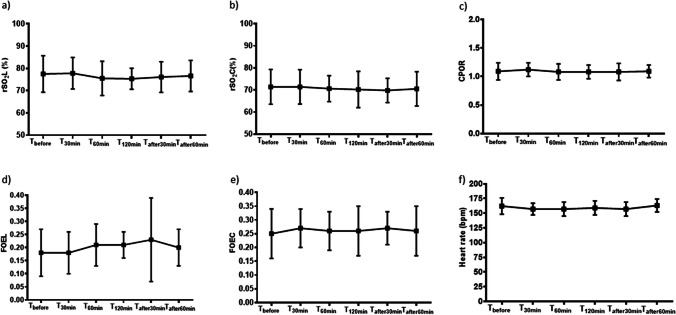
Changes in **a** lung (rSO_2_L) and **b** cerebral (rSO_2_C) oxygenation, **c** lung (FOEL) and **d** cerebral (FOEC) fractional oxygen extraction ratio, **e** cerebro-pulmonary oxygenation ratio (CPOR), and **f** heart rate at the different data points of the study. Mean ± (SD)

Mean values of rSO_2_L, FOEL, rSO_2_C, FOEC, CPOR ratio, and heart rate measured at the different datapoints of the study did not significantly change during the study period. However, we observed a non-statistically significant trend toward a slight decrease in rSO_2_L and increase in FOEL during KMC, as well as a slight decrease in rSO_2_C and increase in FOEC (Fig. 1, Table S2).

**Fig. 2:**
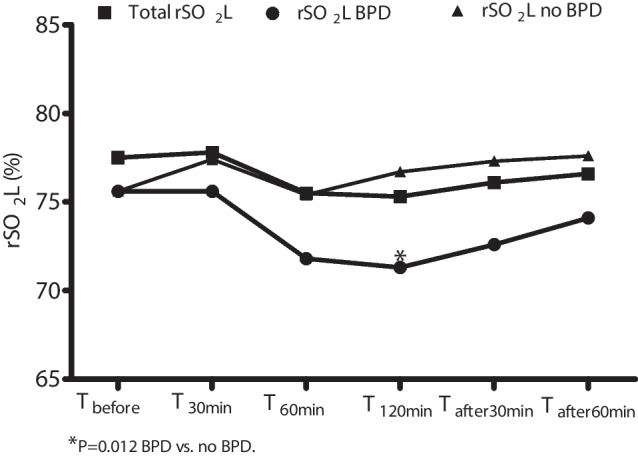
Changes in lung oxygenation (rSO_2_L) in the total population and in infants with or without bronchopulmonary dysplasia (BPD)

Among the subgroups of infants with (*n* = 6; 30%) or without (*n* = 14 = 60%) BPD, we found that rSO_2_L was significantly lower at T_120min_ in the former than in the latter (71.3 ± 1.4 vs. 76.7 ± 4.6%; *P* = 0.012). Conversely, we found that FOEL was significantly higher at T_120min_ in infants with BPD than in infants without it (0.26 ± 0.02 vs. 0.20 ± 0.05; *P* = 0.012). rSO_2_C, FOEC, and CPOR ratio were similar between infants with or without BPD (Fig. 2, Table S3).

## Discussion

In this study, we monitored the lung oxygenation in preterm infants during KMC for the first time and we can confirm the hypothesis that rSO_2_L does not worsen. These data support the knowledge that KMC is safe in preterm infants in stable clinical conditions and that it does not significantly interfere with respiratory function.

It is interesting that we observed a trend toward a slight decrease in lung oxygenation during KMC which was compensated for by a similar slight increase in FOEL. This finding suggests that infants with stable vital parameters can effectively compensate possible postural changes also increasing oxygen blood extraction. However, the values of rSO_2_L that we measured during KMC were slightly higher than those that we measured in preterm infants with RDS [14] or BPD [30]. These slight little changes in lung oxygenation during KMC are clinically insignificant, as also indicated by the contemporary normal values of cerebral oxygenation. These results are in agreement with Demirel et al., who found that clinically stable very low birth weight infants are able to maintain stable cerebral oxygenation in the supine position with the bed tilted upwards by 30° at a mean age of 42.4 ± 15.7 days of life [9], and with Schrod et al., who observed that rSO_2_C did not vary during KMC in preterm infants from 2 to 12 days of life [10].

We found that after 2 h of KMC, infants with BPD had lower rSO_2_L compared to infants without BPD. This decrease was compensated with the increase in FOEL. However, during the study period, lung oxygenation remained in the range of values previously found in preterm infants with BPD in the supine position at similar postnatal age [30]. Considering that none of the infants without BPD required respiratory support at NIRS recording and that the SpO_2_ target was similar in both groups, the different rSO_2_L and FOEL values found in infants with BPD were probably due to the progression of lung injury [31, 32] which negatively influenced lung oxygenation and induced effective compensatory mechanisms.

In our patients, CPOR did not vary during the study period, either in the overall population or in the subgroups of patients with or without BPD. This finding supports the concept that during KMC neither lung nor cerebral perfusion changes and that this procedure does not cause negative cardiorespiratory effects in preterm infants.

The strengths of this study include the originality of data and the effort of stratifying our population in the groups of infants with and without BPD. Although our population is small, these data could be used as a comparison for further studies on the same subject. Limitations include the fact that we did not record the respiratory rate in our infants. However, the stability of heart rate and SpO_2_ during KMC and the fact that it was never discontinued suggest that our patients did not present apnea, tachypnea, and/or dyspnea during KMC. Moreover, we did not thoroughly evaluate the heart rate variability in our patients using methods such as spectral analysis. However, previous studies gave discordant results, showing a decrease in both low- and high-frequency heart rate [33], an increase in low-frequency heart rate alone [34], or an increase in low-frequency and a decrease of high-frequency heart rate [2] during KMC. However, while these data are useful to evaluate the balance between sympathetic and parasympathetic activities, they were not the aim of our study.

In conclusion, we found that rSO_2_L measured by NIRS in preterm infants during KMC did not change, as did rSO_2_C, FOEL, and FOEC. We observed a transient decrease in rSO_2_L compensated for by the increase in FOEL only in infants with BPD, but these changes were clinically insignificant. These results confirm the safety of KMC in preterm infants who are in stable clinical conditions and suggest that KMC does not significantly interfere with cardiorespiratory function even in infants with BPD.

## Supplementary Information

Below is the link to the electronic supplementary material.

Supplementary file1 (DOCX 20 KB)

## Data Availability

Data are available on reasoned request.

## Abbreviations

BPD: Bronchopulmonary dysplasia
CPOR: Cerebro-pulmonary oxygenation ratio
FOEC: Cerebral tissue oxygen extraction fraction
FOEL: Lung tissue oxygen extraction fraction
KMC: Kangaroo mother care
IVH: Intraventricular hemorrhage
NEC: Necrotising enterocolitis
ROP: Retinopathy of prematurity
rSO_2_C: Cerebral oxygenation
rSO_2_L: Lung oxygenation
