# Supplementary material for: Monitoring lung and cerebral oxygenation using near-infrared spectroscopy in preterm infants during kangaroo mother care
Source: Eur J Pediatr. 2024 Aug 9;183(10):4411–6. doi: 10.1007/s00431-024-05674-5 (PMC11413158; doi:10.1007/s00431-024-05674-5)
Supplement: Supplementary file 1 — Supplementary file1 (DOCX 20 KB) [file 431_2024_5674_MOESM1_ESM.docx]

**Table S1.** Respiratory supports, prematurity complications, and maternal disorders of pregnancy in preterm infants studied during Kangaroo mother care (KMC). Mean (+SD) or rate (%).

|  | Infants studied during KMC  (n=20) |
| --- | --- |
| Noninvasive ventilation  Duration (d) | 19  19.3+21.7 |
| Mechanical ventilation  Duration (d) | 3  2.0+5.7 |
| Postnatal steroids | 3 |
| Patent ductus arteriosus | 9 |
| Bronchopulmonary dysplasia  Mild  Moderate  Severe | 6  2  1  3 |
| Necrotizing enterocolitis | 1 |
| Sepsis | 3 |
| 1^st^ degree intraventricular haemorrhage | 0 |
| Retinopathy of prematurity  >3 grade | 2  0 |
| Death | 0 |
| Duration of hospital stay (d) | 67+26 |
| Placental abruption | 1 |
| Gestational diabetes | 4 |
| Hypertensive disorders of pregnancy | 4 |
| Choriamnionitis | 2 |
| pPROM >18 hours | 8 |

pPROM: prolonged premature rupture of membranes

**Table S2.** Changes in lung (rSO_2_L) and cerebral (rSO_2_C) oxygenation, lung (FOEL) and cerebral (FOEC) fractional oxygen extraction ratio, cerebro-pulmonary oxygenation ratio (CPOR), and heart rate at the different data points of the study. Mean+(SD).

|  | **T_Before_** | **T_30min_** | **T_60min_** | **T_120min_** | **T_after30min_** | **T_after60min_** |
| --- | --- | --- | --- | --- | --- | --- |
| **rSO_2_L (%)** | 77.5+8.2 | 77.8+7.1 | 75.5+7.7 | 75.3+4.7 | 76.1+6.9 | 76.6+7.0 |
| **FOEL** | 0.18+0.09 | 0.18+0.08 | 0.21+0.08 | 0.21+0.05 | 0.23+.0.16 | 0.20+0.07 |
| **rSO_2_C (%)** | 71.4+7.9 | 71.4+7.8 | 70.6+5.9 | 70.2+8.2 | 69.8+5.5 | 70.5+7.8 |
| **FOEC** | 0.25+0.09 | 0.27+0.07 | 0.26+0.07 | 0.26+0.09 | 0.27+0.06 | 0.26+0.09 |
| **CPOR** | 1.09+0.15 | 1.12+0.12 | 1.08+0.14 | 1.08+0.12 | 1.08+0.15 | 1.09+0.11 |
| **Heart rate (bpm)** | 162+14 | 157+10 | 157+12 | 159+12 | 157+12 | 163+11 |

**Table S3**. Age at NIRS recording, rSO_2_L, FOEL, SpO_2_/FiO_2_, and a/ApO_2_ in infants with and without BPD. Mean (+SD).

|  | **BPD**  **(n=6)** | **No BPD**  **(n=14)** | **P** |
| --- | --- | --- | --- |
| **Age (d)** | 58+24 | 30+17 | 0.008 |
| rSO_2_L (%) | | | |
| **T_Before_**  **T_30min_**  **T_60min_**  **T_120min_**  **T_after30min_**  **T_after60min_** | 75.6+4.2  75.6+4.1  71.8+6.6  71.3+1.4  72.6+2.4  74.1+2.9 | 75.6+5.1  77.4+5.4  75.4+4.5  76.7+4.6  77.3+7.5  77.6+7.8 | 1.000  0.476  0.171  0.012  0.156  0.306 |
| FOEL | | | |
| **T_Before_**  **T_30min_**  **T_60min_**  **T_120min_**  **T_after30min_**  **T_after60min_** | 0.17+0.11  0.17+0.11  0.21+0.12  0.26+0.02  0.23+0.03  0.23+0.03 | 0.19+0.08  0.19+0.07  0.21+0.05  0.20+0.05  0.19+0.07  0.19+0.08 | 0.652  0.628  1.000  0.012  0.120  0.256 |
| **rSO_2_C (%)** | | | |
| **T_Before_**  **T_30min_**  **T_60min_**  **T_120min_**  **T_after30min_**  **T_after60min_** | 68.6+2.5  69.2+2.4  68.6+3.6  66.2+4.3  67.3+4.0  65.2+2.3 | 72.4+8.4  70.2+6.1  71.2+6.4  71.2+8.9  70.8+5.6  72.1+8.4 | 0.286  0.705  0.503  0.211  0.185  0.067 |
| **FOEC** | | | |
| **T_Before_**  **T_30min_**  **T_60min_**  **T_120min_**  **T_after30min_**  **T_after60min_** | 0.28+0.04  0.27+0.03  0.28+0.03  0.31+0.04  0.29+0.05  0.32+0.03 | 0.24+0.10  0.27+0.08  0.25+0.07  0.25+0.10  0.26+0.07  0.26+0.07 | 0.362  1.000  0.331  0.177  0.357  0.061 |
| **CPOR** | | | |
| **T_Before_**  **T_30min_**  **T_60min_**  **T_120min_**  **T_after30min_**  **T_after60min_** | 1.10+0.07  1.09+0.07  1.05+0.09  1.08+0.06  1.02+0.16  1.14+0.08 | 1.06+0.12  1.11+0.09  1.07+0.10  1.09+0.14  1.10+0.14  1.08+0.12 | 0.460  0.635  0.679  0.870  0.276  0.280 |

*P <0.001 vs 1^st^ and 2^nd^ recording; **P <0.001 vs 1^st^ recording; ***P=0.042 vs 1^st^ recording.
